# The impact of uncertainty on disclosure of prenatal exome sequencing results: A vignette study among medical students

**DOI:** 10.1371/journal.pone.0349014

**Published:** 2026-05-14

**Authors:** Jasmijn E. Klapwijk, Vyne Van der Schoot, Marike G. Polak, Stina Lou, Ida Vogel, Iris M. Jansen-Bakkeren, Karin E. M. Diderich, Katinka Dijkstra, Sam R. Riedijk

**Affiliations:** 1 Erasmus School of Social and Behavioural Sciences, Erasmus University Rotterdam, Rotterdam, The Netherlands; 2 Department of Clinical Genetics, Erasmus Medical Center, Rotterdam, The Netherlands; 3 Center for Fetal Diagnostics, Aarhus University Hospital, Aarhus, Denmark; 4 Department of Clinical Medicine, Aarhus University, Aarhus, Denmark; 5 Department of Clinical Genetics, Aarhus University Hospital, Aarhus, Denmark; Mediterranean University of Reggio Calabria: Universita degli Studi Mediterranea di Reggio Calabria, ITALY

## Abstract

**Introduction:**

We recruited medical students for a study to investigate how they make decisions regarding which hypothetical genetic results to report during pregnancy. We tested how medical students as healthcare professionals handled uncertain prenatal Exome Sequencing results, and whether intolerance of uncertainty (IU) influenced decisions to share results with prospective parents. Exploring the impact of uncertainty on important medical decisions can help optimize counselling.

**Materials and methods:**

Fifty-one participants ranked vignettes covering several types of uncertain prenatal ES results on perceived uncertainty. Participants answered whether they would report each result to the theoretical couple and indicated how certain they were about their choice. Additionally, their IU was measured using the Intolerance of Uncertainty Scale (IUS) and results were compared between low IU and high IU to test whether this influenced the results. The role of termination of pregnancy (TOP) considerations on these variables and experienced anxiety was also explored.

**Results:**

Participants reported fewer highly uncertain results than results low in uncertainty. Participants were more uncertain about their choices to report with highly uncertain results than results low in uncertainty. IU did not influence these relationships. Anxiety of the students when parents considered TOP was not correlated with number of reported results nor certainty with these reporting choices.

**Conclusions:**

In this study we validated the widespread hypothesis that the willingness to convey uncertain prenatal ES results decreased as uncertainty increased. This willingness did not seem to be higher or lower when taking into account IU. More research among experienced healthcare professionals is needed to ascertain whether findings from this study extend to real-life decision-making.

## Introduction

Prenatal DNA testing through Exome Sequencing (ES) enables analysis of the entire exome [1] and has been proven to be a powerful diagnostic tool in case of fetal anomalies [2,3]. Although the diagnostic yield has improved with ES compared to its predecessors, such as karyotyping and microarray, the probability to encounter uncertain results is higher than ever [4,5].

Uncertain information potentially discomforts Health Care Professionals (HCPs) [6], and has been associated with a reluctance to disclose this medical information [7,8]. The extent to which uncertain results should be disclosed to prospective parents has proven to be a difficult matter [9]. Moreover, there is a lack of guidelines (nationally and internationally) to guide HCPs in how to handle these findings [5,9–11]. Due to the lack of guidelines, HCPs in prenatal care may employ varying approaches in dealing with uncertain outcomes of genetic testing [12,13] which may be influenced by characteristics of the HCP.

HCPs bear the responsibility to guide pregnant couples towards informed decisions that align with their values or attitudes [14], while preventing to cause more anxiety and distress. Several aspects have been shown to influence the HCPs decision about disclosure of an uncertain prenatal genetic test result. Causing psychological harm by potentially burdening prospective parents with stress or anxiety, has been identified by HCPs as a major concern [5,15–18]. According to HCPs, personal preferences of the patient were considered important in guiding reporting decisions [16,17], as well as pathogenicity and actionability of the genetic result [19–22]. Parents might consider termination of pregnancy (TOP) when anomalies in their fetus are seen. Some HCPs reported an increase in discomfort and uncertainty when decisions regarding TOP were involved, and expressed to have more doubts about relaying uncertain results [6].

Another aspect that might influence the HCPs decision about disclosure of an uncertain prenatal genetic test result, may be intolerance of uncertainty, either in the parents or in the HCP themselves. Intolerance of uncertainty (IU) was originally suggested as a vulnerability factor for worry and General Anxiety Disorder (GAD) [23] and has played a role in the treatment of anxiety related disorders [24]. Over time, IU started to be considered to be a transdiagnostic and trans-situational construct [23]. This includes the medical setting which can be characterized by high-stress situations where uncertainty will always play a role, and where intolerance of uncertainty has been linked to negative outcomes for HCP’s [25]. For example, higher levels of IU among HCP’s have been linked to negative effects in the investigation process (e.g., withholding information, more referrals to peers) and in prescription behaviours (e.g., overprescribing tests) [26].

In regard to the influence of IU specifically on medical professionals disclosing uncertain information, one study demonstrated that when an HCP perceived their patient to have a higher intolerance of uncertainty they were less likely to want to communicate ambiguous information to them [7]. Likewise, a study using tolerance of ambiguity, a construct closely related to uncertainty, found that HCPs who had a lower tolerance for ambiguity were less inclined to disclose unfavorable genetic test results [27]. Intolerance of uncertainty of an HCP may thus directly or indirectly impact a patients’ decision-making process [28].

Yet, studies looking into the effect of HCP characteristics like (in)tolerance of uncertainty on behavior and/or decision-making among HCPs are limited, especially in the prenatal setting which has different challenges (e.g., termination of pregnancy) than postnatal or more general medical settings. Understanding how (intolerance of) uncertainty influences the HCPs’ inclination to convey ambiguous information may enable the development of guidelines to support HCPs in making well-considered choices regarding uncertain prenatal ES results and may serve to offer adequate counseling to pregnant couples. In the current study we therefore aimed to gain insights among medical students into the effect of uncertainty and intolerance of uncertainty on HCP decisions to disclose uncertain genetic test results within the prenatal context.

## Materials and methods

### Participants

We included fifth year medical students who were at the start of their gynecology and pediatrics internship (*N* = 53) from the Erasmus Medical Center in Rotterdam. This internship is part of the standard curriculum. There is only a small, limited number of clinical geneticists in the Netherlands. A convenience sample of medical students was chosen, because conducting this study among students afforded us to gain insights among a less experienced, but larger sample size. During their Bachelor studies, all these students have been taught extensively about genetics, as the Erasmus medical curriculum is genetics heavy. In this medical cohort, female students are overrepresented. Two participants were excluded due to not completing the ranking task, leaving a final sample size of 51. Participants were proficient in Dutch, between 22 and 43 years old (*M* = 24.75, *SD* = 3.03) and mostly female (70.6%). Participation was on a voluntary basis. Testing took place between 25-4-2019 and 10-07-2019 and informed written consent was obtained from each participant. The study protocol (MEC-2019–0019) was assessed by the Medical and Ethical Assessment Committee (METC) of the Erasmus Medical Center and exempted from ethical approval as the act of Medical Research involving Human Subjects (WMO) did not apply.

### Procedure

Participants were primed for their role as clinical geneticists by recruiting them during a (prenatal) genetic counselling class. After signing up, participants received a link to the survey in Qualtrics (https://www.qualtrics.com) via e-mail.

First, we described a case presenting a pregnant couple who was confronted with a fetal abnormality on the 20-week ultrasound. Participants were asked to place themselves in the role of genetic healthcare professionals employing exome sequencing (ES) to investigate a potential genetic cause. Eight vignettes with different results were presented in randomized order and participants answered whether they would report the result to the pregnant couple or not. However, one vignette featured the option to employ an unfiltered ES (instead of a targeted ES). For this question only, participants answered two questions: 1) whether the participant would want to look at the unfiltered ES if they were the clinical geneticist, and 2) whether they would present the option to employ an unfiltered ES to the pregnant couple. For all vignettes, participants then indicated how certain they were of their decision on a Visual Analog Scale (VAS; a slider ranging from 0 to 100) where 0 represented ‘Not at all sure’ and 100 represented ‘Absolutely sure’. The slider was set at 50 as a default and the slider had to be actively moved by the participant to be able to continue. Similarly, participants indicated how much anxiety (0 = No anxiety, 100 = A lot of anxiety) they experienced if the pregnant couple indicated they were considering terminating the pregnancy based on the result they would receive.

For the second task, participants ranked a list of shortened versions of the vignettes (randomized) based on how much uncertainty the vignettes elicited as perceived by the participant (1 = Most uncertain, 9 = Least uncertain). Thirdly, participants completed the (Intolerance of Uncertainty Scale (IUS) [29,30] and then answered demographic questions. Completion of the survey took approximately 15 minutes.

### Materials and design

#### Vignettes

Vignettes were developed based on identified uncertainties in prenatal ES. We used types of uncertainties, and their definitions as described in the paper by [11]. Subsequently, we conducted interviews with 31 HCPs to identify which uncertainties they perceived as being problematic and to ask about their experiences with these uncertainties and the influence on their decision-making process. A more elaborate report of these interviews and the results they yielded was published elsewhere [31]. We then developed eight vignettes, each addressing an uncertainty type (Table 1).

**Table 1:** Framework for the Content of the Vignettes.

Vignette topic
**1. Clear ultrasound. Shorter than 1.40m, no intellectual disability.** The ultrasound has found that the bones in all limbs are clearly too short. ES has found a genetic cause. For this genetic cause, it is known that carriers cannot grow beyond 1.40m and this genetic cause is not associated with other abnormalities (e.g., no intellectual disability).
**2. Incidental finding (fetus):** The ultrasound has found that the bones in all limbs are too short. The abnormality is bordering between normal and deviating. ES has found a genetic defect, but it does not cause the bone abnormality. The genetic defect increases the risk of arrhythmia. The child will have to have a check-up with the cardiologist once every 2 years from childhood onwards. When cardiac arrhythmia develops, it will be easily treated with a pacemaker.
**3. Ambiguous ultrasound. Shorter than 1.40m, no intellectual disability.** The ultrasound has found that the bones in all limbs are too short. The abnormality is bordering between normal and deviating, but both parents are just above average in height. ES has found a genetic cause. For this genetic cause, it is known that carriers cannot grow beyond 1.40m and this genetic cause is not associated with other abnormalities (e.g., no intellectual disability).
**4. Clear ultrasound. Variant of Uncertain Significance.** The ultrasound has found that the bones in all limbs are clearly too short. ES has found a genetic abnormality, but it is not clear whether it explains the shortened limbs. It is also not known whether this variant will cause other problems in this child.
**5. Incidental finding (parent).** The ultrasound has found that the bones in all limbs are too short. The abnormality is bordering between normal and deviating, but the father is taller than average, and the mother has an average height. The fetus’ and both parents’ ES (trio-analysis) shows that the mother is carrier of a mosaic Turner syndrome, and the fetus, a daughter, has Turner syndrome (full mutation). The mother has to be monitored for her heart, and there’s a chance she will go into menopause early.
**6. Ambiguous ultrasound. Variant of Uncertain Significance (VUS).** The ultrasound has found that the bones in all limbs are too short. The abnormality is bordering between normal and deviating, but both parents are just above average in height. ES has found a genetic abnormality, but it is not clear whether it explains the shortened limbs. It is also not known whether this variant will cause other problems in this child.
**7. Variable expression.** The ultrasound has found that the bones in all limbs are too short. The abnormality is bordering between normal and deviating, but both parents are just above average in height. ES has found a genetic abnormality. This genetic cause has a variable expression: carriers are usually smaller than average, but not always; about 1 in 2 carriers has an intellectual disability ranging from mild learning disability to severe intellectual disability. This means that the child will hardly have any complaints after birth but can also be so seriously affected that it will never be able to function independently. The severity of the intellectual disability cannot be predicted during pregnancy.
**8. Unfiltered ES.** The ultrasound has found that the bones in all limbs are too short. The abnormality is bordering between normal and deviating, but both parents are just above average in height. When using ES, a filter was used that only looked at the genes that are known to cause bone abnormalities. No cause has been found this way. The clinical geneticist has the option to view the ES results unfiltered. This increases the chance to find a genetic cause for the bone defect after all, but also increases the chance of encountering a genetic abnormality with unclear meaning.

These vignettes were adjusted by a medical psychologist (SR) experienced in counselling couples impacted by prenatal genetic testing to ensure vignettes represented real-life situations. Subsequently, the vignettes were checked by an experienced clinical geneticist (LG) to ensure the anomalies that were presented in the vignettes accurately matched possible ES findings. All vignettes were presented in Dutch, the participants’ native language (see S1 File for translated vignettes). Lastly, vignettes were pilot tested among four graduate students with proficient knowledge in clinical genetics. They tested the readability and representation of the survey, after which we made minor changes in wording (leading to the vignettes displayed in Table 1).

### Uncertainty and anxiety

Participants indicated whether they would report the ES result presented in each vignette or not, and then indicated certainty with their choice on a VAS-scale (0–100 slider).

Anxiety when the decision to terminate the pregnancy (TOP) was involved, was measured through a VAS-scale. Lastly, participants ranked the vignettes on perceived uncertainty (1 = most uncertain, 8 = least uncertain) (S1 File).

### Questionnaires

We used the Dutch translation of the Intolerance of Uncertainty Scale – Short form (IUS-12 Dutch, *α = .83*) [29,30] to measure participants’ intolerance of uncertainty (see S1 File). Total IU scores were calculated by summing the scores of all twelve statements. A higher score represented a higher intolerance of uncertainty.

### Analyses

**Fig 1:**
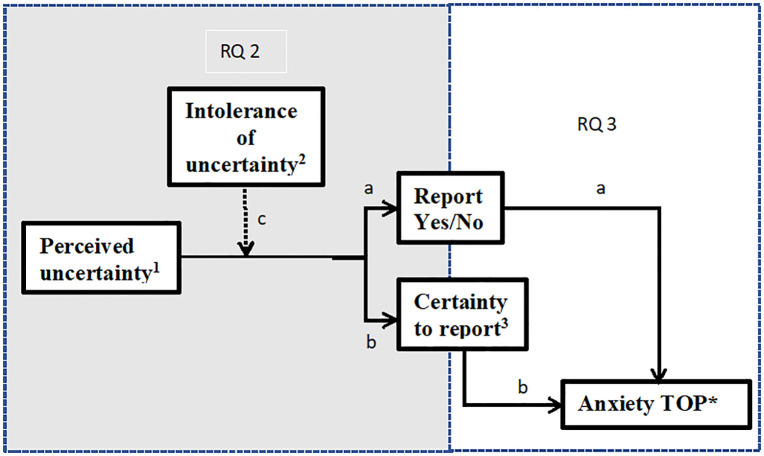
Model of the experiment split by research questions. 1 Perceived uncertainty measured by the ranking of uncertainty of the vignettes (1 = Most uncertain, 9 = Least uncertain). 2 Intolerance of uncertainty as measured by the IU-scale [29]. Total score ranging from 12–60. 3 Certainty with choice to report the result or not as measured on a VAS-scale, 0 = No uncertainty at all, 100 = Very certain. *TOP = Termination of Pregnancy.

Fig 1 shows the research questions we investigated. Our first research question was to find out to what degree the level of uncertainty of the vignettes influenced the participants’ decision to report the result and to elucidate the role of intolerance of uncertainty on this decision-making process. We hypothesized that participants would be more reluctant to report results high in uncertainty than results low in uncertainty. We used McNemar tests to determine whether the proportions of choices to report or not were different between the levels of uncertainty. We hypothesized that intolerance of uncertainty (IU) would influence decision-making. Specifically, high uncertainty would lead to even less willingness to report back highly uncertain results among participants with higher scores of IU. The influence of IU on reporting choices was tested by using a median split on the IU scores. Exact McNemar tests were then performed separately for low IU scores and high IU scores.

Furthermore, we aimed to find out whether participants would report either more or less uncertainty making the choice to report or not depending on the level of uncertainty of the vignette. We hypothesized that IU would influence self-reported certainty about the decisions, with high uncertainty of the vignette leading to more self-reported uncertainty among those with higher scores of IU. We used a mixed ANOVA with self-reported certainty associated with the choice as a dependent variable and the level of uncertainty of the vignettes as a within-subjects factor. To investigate the role of intolerance of uncertainty, level of IU (median split) was added as a between-subjects factor.

Lastly, we explored the influence of termination of pregnancy (TOP) considerations. We measured whether the degree of self-reported anxiety by the medical student when a prospective parent indicated to consider TOP was influenced by 1) the number of times participants chose to report a result, and/or 2) by certainty with the decision made. We used correlations to investigate whether these variables were related. Fig 1 illustrates the last two questions and the relationships we investigated.

## Results

Sixty-two out of 112 medical students (55%) signed up to participate. Fifty-three students (85%) fully completed the experiment. Due to technical issues, no ranking of vignettes on perceived uncertainty was obtained from two participants. Data from these participants were excluded from further analyses, resulting in a final sample size of 51. See Table 2 for an overview of the outcome variables.

**Table 2:** Perceived Levels of Uncertainty, Percentages of Reporting Choices, Certainty with Reporting Choices and Anxiety with TOP Considerations per Vignette.

Vignettes^1^ (from most uncertain, to least uncertain)	Median ranking	Perceived uncertainty	% Report *N* = 51	Certainty to Report *M* (*SD*)	Anxiety TOP *M* (*SD*)
V6: VUS, amb.	2	High	68.6%	72.69 (21.57)	69.14 (24.58)
V8: Unfiltered ES	3	High	80.4%	77.80 (20.92)	61.75 (24.60)
V7: Variable expression	3	High	100%	89.47 (14.27)	46.98 (23.27)
V4: VUS, clear	4	Moderate	74.5%	68.94 (23.99)	64.49 (23.80)
V2: IF (fetus)	5	Moderate	98%	87.04 (16.17)	72.78 (25.09)
V3: < 1.40m, no ID, amb.	6	Low	98%	89.61 (16.08)	66.65 (27.23)
V5: IF (parent)	6	Low	100%	91.69 (13.71)	46.98 (25.31)
V1: < 1.40m, no ID, clear	7	Low	100%	89.71 (15.38)	66.04 (27.85)

^1^ In order of uncertainty elicited by the vignettes as ranked by the sample of participants.

Median ranking = The median score of uncertainty for each vignette as ranked by the sample of participants.

Perceived uncertainty = Qualitative subdivision of the vignettes into low, moderate, and high uncertainty, based on the median.

% Report = The percentage of participants who indicated that they would report the genetic abnormality for a vignette.

Certainty to Report = The amount of self-reported certainty when deciding to report the vignette or not (0–100). A higher score reflects more certainty.

Anxiety TOP = The amount of anxiety experienced by the medical student when deciding to report the vignette or not, when accounting for the notion that the pregnant couple would consider terminating the pregnancy (TOP) based on the vignette (0–100).

### Reporting choices

Table 2 shows that the percentage of participants who would report the genetic abnormality described in each vignette varied from 68.6% (V6. VUS, amb) to 100% (V7. Variable expression, V5. IF parent and V1. < 1.40m, no ID, clear).

**Fig 2:**
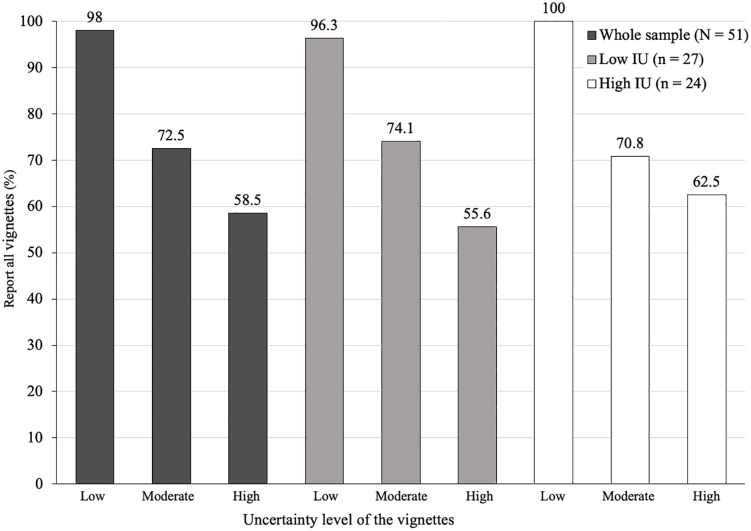
Choices to report the genetic abnormality for all vignettes of a given uncertainty for the whole sample, low IU participants and high IU participants.

Fig 2 shows for each vignette type (perceived uncertainty: low, moderate, high) which percentage of participants (*N* = 51) indicated to report the genetic abnormality for all vignettes of that uncertainty level and which percentage of participants would *not* report the genetic abnormality for all vignettes of that uncertainty level, also split per IU level (mean split).

The uncertainty level of the vignettes was related to the choice to report the genetic abnormality, with higher uncertainty results being related to being less likely to be reported. This relation was not associated with IU of the student, since participants with low and high IU showed similar patterns of results. McNemar tests for the whole sample (*N* = 51) showed that differences between percentages of participants indicating to report all vignettes differed between vignettes with high uncertainty (58.5%) and low uncertainty (98%), as well as between vignettes with moderate uncertainty (72.5%) and low uncertainty (98%) with, respectively, χ^2^ (1, *N* = 51) = 18.05, *p* < .001 and χ^2^ (1, *N* = 51) = 11.08, *p* < .001. Whereas the proportion of participants reporting all moderate uncertainty vignettes (72.5%) and the proportion of participants reporting all high uncertainty vignettes (58.5%) did not differ significantly, χ^2^ (1, *N* = 51) = 2.77, *p* = .092. These results were similar for participants low in IU and participants high in IU (see statistics in S2 File), including the significance of pairwise differences.

Correlational analyses with IU total scores confirmed the above findings. The correlation between IU total scores and the total number of vignettes reported was non-significant, *r*(49) =.01, *p* = .96. Similarly, when Spearman rank-order correlations were computed between IU total scores and the total number of vignettes reported separately for low-, moderate-, and high-uncertainty vignettes, no significant associations were observed (*r*_*s*_ = .03, *p* = .81; *r*_*s*_ = .01, *p* = .94; *r*_*s*_ = .00, *p* = .98, respectively).

### Degree of certainty of disclosure

In the following analysis we investigated whether certainty to report differed between vignette types (perceived uncertainty: low, moderate, high) and whether differences were associated with intolerance of uncertainty (IU: low, high). High and moderate uncertainty vignettes elicited less certainty to report than low uncertainty vignettes, irrespective of IU level.

**Fig 3:**
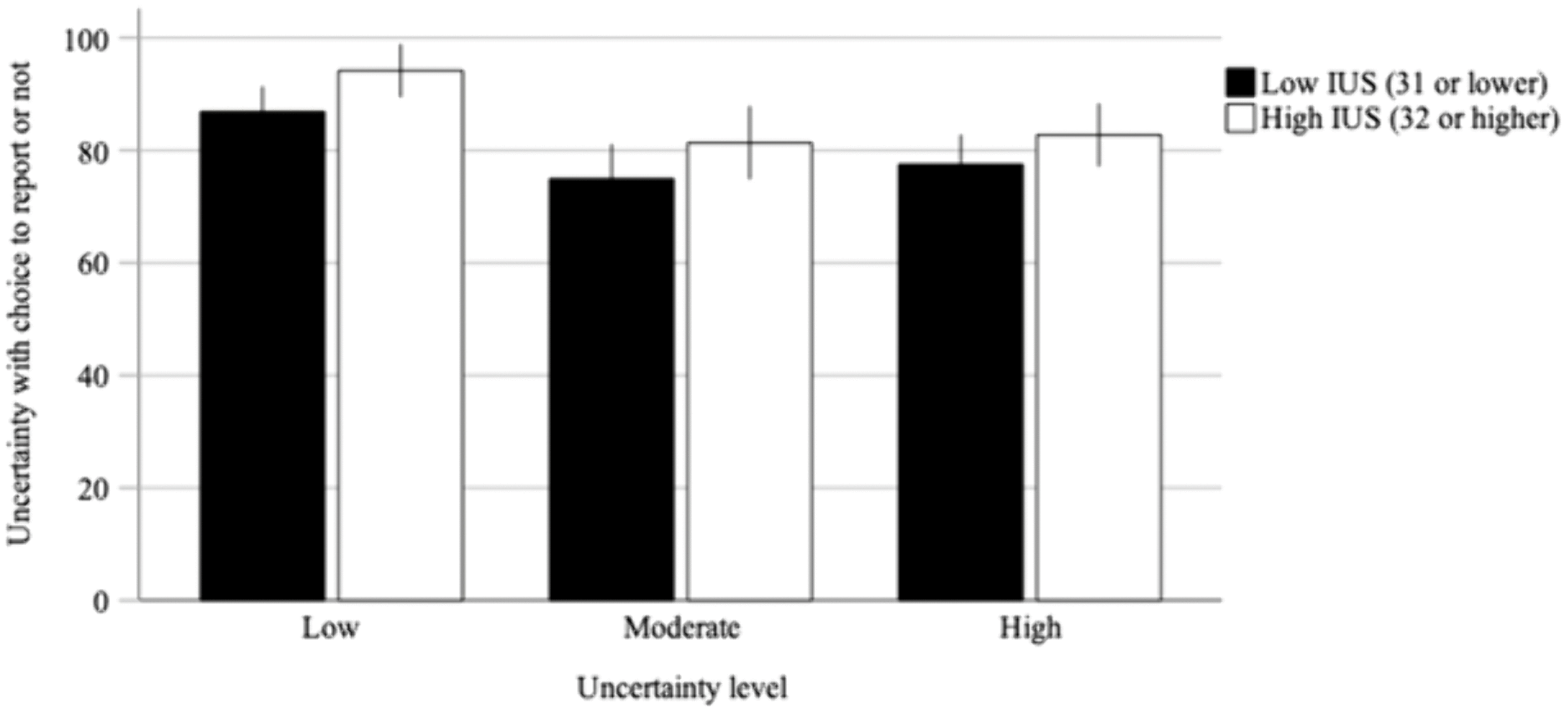
Self-reported certainty with choices to report or not per uncertainty level of the vignettes separated by level of IU (low or high). The error bars display the 95% CI.

Fig 3 shows the self-reported certainty of participants’ choices to report or not per uncertainty level of the vignettes separately for different IU levels (low and high). A 2 (low or high IU) x 3 (low, moderate, or high uncertainty level of the vignettes) mixed ANOVA showed that, overall, participants with high IU (*M* = 86.70, *SD* = 8.60) were more certain to report than participants with low IU (*M* = 80.41, *SD* = 11.93), *F*(1, 49) = 4.32, *p* = .043, *η*^*2*^ = .08. Furthermore, participants’ certainty to report differed between vignette types, *F*(2, 98) = 21.44, *p* < .001, *η*^*2*^ = 0.30, where participants reported less certainty to report for vignettes with high uncertainty (*M* = 79.99, *SD* = 13.65) than for vignettes with low uncertainty (*M* = 90.33, *SD* = 11.85), *t*(50) = 5.11, *p* < .001. Likewise, par*t*icipants reported less certainty to report when vignettes were moderately uncertain (*M* = 77.99, *SD* = 15.90) compared to vignettes with low uncertainty (*M* = 90.33, *SD* = 11.85), *t*(50) = 5.37, *p* < .001. Moderately uncer*t*ain vignettes (*M* = 77.99, *SD* = 15.90) did not differ from highly uncertain vignettes (*M* = 79.99, *SD* = 13.65) in participants’ certainty with their choices to report or not, *t*(50) = 1.15, *p* = .766. Finally, it can be seen in Fig 3 tha*t* differences between vignette types regarding participants’ certainty to report were not related to level of IU, *F*(2, 98) = 0.13, *p* = .883, *η*^*2*^ = 0.00. That is, differences between vignette types were similar for low and high IU, although overall participants high in IU were more certain to report than participants with low IU.

### TOP considerations

Lastly, we explored anxiety in response to prospective parents considering termination of pregnancy (TOP) (see Table 2 for mean anxiety score per vignette). There did not seem to be a clear relation between self-reported anxiety and number of results reported and certainty to report.

Correlations were investigated between self-reported anxiety in response to prospective parents considering termination of pregnancy (TOP) and 1) number of vignettes for which results were reported and 2) certainty to report. Self-reported anxiety scores were missing for one participant leaving a sample size of *n* = 50 for the following analyses. The number of reported results was not significantly correlated with self-reported anxiety, *r*(48) = −.13, *p* = .357. Nor was certainty to report significantly correlated with self-reported anxiety, *r*(48)=.02, *p* = .898.

## Discussion

The current study investigated how differing levels of uncertainty predicted the willingness to convey uncertain prenatal ES results and to assess the influence of (in)tolerance of uncertainty as an HCP characteristic on the decision to disclose uncertain genetic test results in the prenatal context; a context that inherently involves uncertainties.

This study indicated that intolerance of uncertainty did not influence choices to convey uncertain test results; it was mainly the uncertainty of the result that affected the experienced uncertainty. Overall, we observed a high willingness to report results to parents. Participants were less certain about their decision to disclose test results when these were of a more uncertain nature.

### Uncertainty of the results

Uncertainty of a result influenced reporting decisions. In general, higher uncertainty results were chosen less often to be reported than results ranked as low in uncertainty. Likewise, participants were more uncertain about their choices to report with higher uncertainty results than low uncertainty results. Being uncomfortable with uncertain information has been linked before to a lower willingness to disclose medical information [7,8]. In the prenatal setting this may be unfavorable, as some uncertain results may prove to be(come) important. VUS, IF’s and SFs for instance could later turn out to be relevant [32]. A solution could be to discuss uncertain results within a multidisciplinary team (MDT) to be able to decide which to report [32]. Combining different perspectives and expertise may prove helpful in mitigating the risk of personality traits influencing the decision whether or not to disclose results and balancing factors that have been shown to be important in decision-making (e.g., preventing psychological harm [15,18], patients’ preferences [16,17], and pathogenicity of results [21].

Interestingly, vignettes with moderate uncertainty elicited the same reporting choices as high uncertainty vignettes. The self-reported certainty with these choices was also similar for moderate and high uncertainty vignettes. A possible consequence might be that results that may be perceived as ‘only’ moderately uncertain, could provoke underestimated uncertainty and/or anxiety in HCPs and parents. In our analyses, we based our categorization of perceived uncertainty on the median of the rankings (see Table 2). Future research could investigate other ways of categorizing or measuring uncertainty.

Notably, one vignette (V7. Variable expression) that was ranked as highly uncertain did not seem to negatively influence reporting choices as it was reported by all participants. A variant with variable expression will affect the child, but it is uncertain to what extent and what implications it would have for the child once born. From the HCP´s perspective, a clearly pathogenic and causal variant was found. This could indicate that for (student) HCPs the variant’s pathogenicity is an important modifier in the degree of perceived uncertainty. This is in line with studies that demonstrated there is consensus among HCPs that pathogenic and actionable findings should be reported [19–22]. However, a variant with variable expression may cause substantial uncertainty for the prospective parents [33,34]. This uncertainty is highly relevant in the prenatal context as parents will be presented with choices, including about continuing pregnancy, not knowing how the variant will affect their child after birth and without having much phenotypical information to rely on [34]. Awareness of the potential difference in how parents and HCPs experience uncertain results is of great importance for decision counseling [35].

### Intolerance of uncertainty

The relationship between reporting choices and the uncertainty level of the vignettes did not seem to be influenced by intolerance of uncertainty (IU) of the participants. This finding is contrary to the findings of one study that demonstrated HCPs were less inclined to communicate negative genetic test results if they had a lower tolerance for ambiguity [27] and other studies that have found similar negative consequences of intolerance of uncertainty on clinical behavior [26]. The interaction between prospective parents and HCP during genetic counselling is essential for psychological coping and well-being [36]. How uncertainty is handled by patients affects how information is presented [37]. Perhaps IU only influences HCPs decision-making when they perceive their patients to be highly intolerant of uncertainty [7]. Studies on the interaction between patients and HCPs when faced with uncertain results seem essential for the support HCPs can offer. It could also be interesting to see whether these results are specific for this young HCP sample and if so, why that might be. On the one hand, it is preferable to minimize the influence of personal HCP traits (like IU) on counseling. On the other, if IU doesn’t influence young HCP’s, is it possible that uncertainty is not sufficiently activated within the educational setting? If so, this may have implications for teaching good counseling and teaching young HCP’s how to navigate uncertainty (including their own) appropriately.

IU had an independent effect on the certainty with which the choices to report or not were made. Surprisingly, participants that scored high in IU reported more certainty with their choices than participants with low IU, irrespectively of the level of uncertainty of the vignettes. This could be due to variables that were not measured in this study such as reaction time; participants with high IU might have spent more time thinking about their responses, managing their own uncertainty. Future research could evaluate a relation between reaction time and participants’ self-reported certainty levels.

### Influence of TOP considerations

In the interviews during survey-development, some HCPs mentioned that potential considerations involving TOP by prospective parents, provoked anxiety [31]. Although self-reported anxiety of the participants in the face of TOP considerations by the prospective parents was moderately high, this anxiety did not seem to be influenced by experienced uncertainty to report the results. However, these were hypothetical vignettes. The emotions, especially among parents, involved in real-life, high-stakes situations likely magnify the experienced uncertainty and the consequences they have for decision-making [37]. The interaction between patients and HCPs is an important element of the counselling process [36]. In this hypothetical vignette study, the absence of the patient-HCP interaction will have impacted these results.

## Strengths and limitations

One key strength of this study pertains to the rigorous process of survey development that preceded testing. In addition to interviews, uncertainties were identified and definitions developed by an international team of various clinical genetic experts. Furthermore, recruiting medical students as participants for this study enabled us to test our vignettes among a larger sample than would have been possible among experienced genetic HCPs.

One limitation is that the situations described in the vignettes were hypothetical. Interviews were conducted with HCPs [31] to develop vignettes that represented real-life scenarios. However, results may be different in a real-life, high stakes setting. Characteristics of the participants investigated in this experiment can also be considered a limitation of this study. Although participants were in their fifth year and were considered young HCPs and primed in their roles after having followed a genetic counseling class, they were not experienced in clinical genetics. Other studies have demonstrated that HCPs in a later stage of their career may show different response patterns than trainees or HCPs at the start of their careers [38–40]. Replicating this experiment among experienced HCPs should elucidate whether the findings generalize to experienced HCPs.

## Conclusion

Uncertain results in prenatal exome sequencing would have been reported by most of our participants with a difference in perceived certainty of reporting. IU or TOP considerations did not drive reporting choices. Follow-up research should elucidate whether these conclusions generalize to experienced HCPs.

## Supporting information

S1 File Materials. (DOCX)

S2 File McNemars per IU level. (DOCX)
