# Supplementary material for: The impact of uncertainty on disclosure of prenatal exome sequencing results: A vignette study among medical students
Source: PLoS One. 2026 May 14;21(5):e0349014. doi: 10.1371/journal.pone.0349014 (PMC13175358; doi:10.1371/journal.pone.0349014)
Supplement: S1 File — (DOCX) [file pone.0349014.s001.docx]

**S1 File. Materials.**

**Qualtrics Questionnaire**

Thank you for participating in this study. Participation takes approximately 15 minutes. We will ask you questions based on a case. These are questions that clinical geneticists are currently struggling with. Using whole exome sequencing for prenatal research can detect more causes of abnormalities, but also produces more uncertain results. There is no consensus yet on which of these results we should report back to patients. In this survey we ask you what you would do if you were a clinical geneticist. Would you report back the results or not? And what feelings or thoughts do you have when deciding? Now read the case and answer the questions by clicking on the arrow below.

**Case**

A couple of 23 and 26 years old is pregnant for the first time. Everything went well so far, although the woman has been affected by nausea and fatigue. On the 20-week ultrasound, an abnormality is detected: The bones of the fetus are too short in all limbs. This bone malformation can have a variety of genetic causes. In some cases, it’s only associated with a bone malformation, without other issues arising. Other times genes can be involved that cause a syndrome that is associated with intellectual disability. To investigate a potential genetic cause, the clinical geneticist employs *exome sequencing* (ES).

We will present you with 8 different ES results, some of which differ only subtly from each other. So read them carefully and consider what you would decide in each of these cases if you were the clinical geneticist.

**Vignette 1**

The ultrasound has found that the bones in all limbs are clearly too short. ES has found a genetic cause. For this genetic cause, it is known that carriers cannot grow beyond 1.40m and this genetic cause is not associated with other abnormalities (e.g. no intellectual disability).

**Vignette 2**

The ultrasound has found that the bones in all limbs are too short. The abnormality is bordering between normal and deviating. ES has found a genetic defect, but it does not cause the bone abnormality. The genetic defect increases the risk of arrhythmia. The child will have to have a check-up with the cardiologist once every 2 years from childhood onwards. When the cardiac arrhythmia develops, they will be easily treated with a pacemaker.

**Vignette 3**

The ultrasound has found that the bones in all limbs are too short. The abnormality is bordering between normal and deviating, but both parents are just above average in height. ES has found a genetic cause. For this genetic cause, it is known that carriers cannot grow beyond 1.40m and this genetic cause is not associated with other abnormalities (e.g. no intellectual disability).

**Vignette 4**

The ultrasound has found that the bones in all limbs are clearly too short. ES has found a genetic abnormality, but it is not clear whether it explains the shortened limbs. It is also not known whether this variant will cause other problems in this child.

**Vignette 5**

The ultrasound has found that the bones in all limbs are too short. The abnormality is bordering between normal and deviating, but the father is taller than average, and the mother has an average height. The fetus’ and both parents’ ES (trio-analysis) shows that the mother is carrier of a mosaic Turner syndrome, and the fetus, a daughter, has Turner syndrome (full mutation). The mother has already been affected and has to be monitored for her heart, and there’s a chance she will go into menopause early.

**Vignette 6**

The ultrasound has found that the bones in all limbs are too short. The abnormality is bordering between normal and deviating, but both parents are just above average in height. ES has found a genetic abnormality, but it is not clear whether it explains the shortened limbs. It is also not known whether this variant will cause other problems in this child.

**Vignette 7**

The ultrasound has found that the bones in all limbs are too short. The abnormality is bordering between normal and deviating, but both parents are just above average in height. ES has found a genetic abnormality. This genetic cause has a variable expression: carriers are usually smaller than average, but not always; about 1 in 2 carriers has an intellectual disability ranging from mild learning disability to severe intellectual disability. This means that the child will hardly have any complaints after birth but can also be so seriously affected that it will never be able to function independently. The severity of the intellectual disability cannot be predicted during pregnancy.

**Questions for Vignette 1-7**

- Would you want to report this result? (Yes, no)
- How sure are you of this decision? (0= Not at all sure, 100= Absolutely sure)
- What do you experience when the pregnant couple indicate they are considering termination of pregnancy (TOP) based on this result? (0 = No anxiety, 100 = A lot of anxiety)

**Vignette 8**

The ultrasound has found that the bones in all limbs are too short. The abnormality is bordering between normal and deviating, but both parents are just above average in height. When using ES, a filter was used that only looked at the genes that are known to cause bone abnormalities. No cause has been found this way. The clinical geneticist has the option to view the ES results unfiltered. This increases the chance to find a genetic cause for the bone defect after all, but also increases the chance of encountering a genetic abnormality with unclear meaning.

- Would you opt for unfiltered ES? (Yes, no)
- How sure are you of this decision? (0= Not at all sure, 100= Absolutely sure)

**Ranking task**

“Below you will find shortened versions of the results that you have just read. Sort the results by amount of uncertainty by dragging them to the position of your choice. 1 = most uncertain result, 8 = least uncertain result.”

- Bones in all limbs are clearly too short. Genetic defect found; child will not be taller than 1.40m. No other deviations. *[V1. Clear ultrasound, pathogenic finding]*

- Bones in all limbs are too short, bordering between normal and abnormal. Genetic abnormality found that does not cause the bone abnormality, but increased risk of (treatable) heart rhythm disorders. *[V2. IF fetus]*

- Bones in all limbs are too short, bordering between normal and abnormal. Genetic defect found; child will not be taller than 1.40m. No other deviations. *[V3. Amb. ultrasound, pathogenic finding]*

- Bones in all limbs are clearly too short. Genetic defect found, but it is not clear whether it explains the bone defect or whether it will cause other problems. *[V4. Clear ultrasound, VUS]*

- Bones in all limbs are too short, bordering between normal and abnormal. Genetic defect found; Turner syndrome (complete mutation). So, the mother is also unexpectedly affected. *[V5. IF mother]*

- Bones in all limbs are too short, bordering between normal and abnormal. Genetic abnormality found, but it is not clear whether it explains the shortened limbs. It is also not known whether this variant will cause other problems in this child. *[V6. Amb. ultrasound, VUS]*

- Bones in all limbs are too short, bordering between normal and abnormal. Genetic abnormality found with variable expression. It is not clear how tall the child can grow and whether he will be mild or severely mentally disabled. *[V7. Variable expression]*

- Bones in all limbs are too short, bordering between normal and abnormal. No genetic cause found, possibility to use unfiltered ES to investigate further. *[V8. Unfiltered ES]*

**Questionnaires**

**Intolerance of Uncertainty Scale; IUS [25]**.

Initials/ID #:__________________

Date:___________________

The questions below are about how you view uncertainties. Keep the context of the case in mind and your role as a clinical geneticist. There are no right or wrong answers.

###### Please circle the number that best corresponds to how much you agree with each statement.

|  | Not at all characteristic of me | A little characteristic of me | Somewhat characteristic of me | Very characteristic of me | Entirely characteristic of me |
| --- | --- | --- | --- | --- | --- |
| 1. Unforeseen events upset me greatly. | 1 | 2 | 3 | 4 | 5 |
| 2. It frustrates me not having all the information I need. | 1 | 2 | 3 | 4 | 5 |
| 3. Uncertainty keeps me from living a full life. | 1 | 2 | 3 | 4 | 5 |
| 4. One should always look ahead so as to avoid surprises. | 1 | 2 | 3 | 4 | 5 |
| 5. A small unforeseen event can spoil everything, even with the best of planning. | 1 | 2 | 3 | 4 | 5 |
| 6. When it’s time to act, uncertainty paralyses me. | 1 | 2 | 3 | 4 | 5 |
| 7. When I am uncertain I can’t function very well. | 1 | 2 | 3 | 4 | 5 |
| 8. I always want to know what the future has in store for me. | 1 | 2 | 3 | 4 | 5 |
| 9. I can’t stand being taken by surprise. | 1 | 2 | 3 | 4 | 5 |
| 10. The smalles doubt can stop me from acting. | 1 | 2 | 3 | 4 | 5 |
| 11. I should be able to organize everything in advance. | 1 | 2 | 3 | 4 | 5 |
| 12. I must get away from all uncertain situations. | 1 | 2 | 3 | 4 | 5 |

Score:______

Original: Carleton, Norton, & Asmundson, 2007

Translation: Helsen, Van den Bussche, Vlaeyen, & Goubert, 2013

**Control Questions**

- Indicate to what extent the following statements apply to you (1 = Not at all, 5 = always):
  - I have thought about having children.
  - I have thought about having myself tested genetically.
  - I have thought about having my (future) child tested genetically.
- Do you have children? (Yes + how many, No, I do not want to answer this)
- Were you tested genetically? (Yes, No, I do not want to answer this)

**Demographic Questions (Optional)**

- How old are you?
- What is your gender? (Male, Female, Other)
- In which city/municipality have you lived most of your life? (Open-ended)
- What is your nationality? (Open-ended)
- What is your religion? (Open-ended)
