# Supplementary material for: The impact of uncertainty on disclosure of prenatal exome sequencing results: A vignette study among medical students
Source: PLoS One. 2026 May 14;21(5):e0349014. doi: 10.1371/journal.pone.0349014 (PMC13175358; doi:10.1371/journal.pone.0349014)
Supplement: S2 File — (DOCX) [file pone.0349014.s002.docx]

**S2 File. McNemars per IU level.**

To investigate whether intolerance of uncertainty (IU) influenced reporting decisions, a median split on the IU scores was used. Within the range of 12-60, a score of 31 or lower formed the low IU category and a score of 32 or higher formed the high IU category. The exact McNemar tests were repeated per category (low and high IU). Table 3 shows the number of participants reporting everything versus not reporting everything for the participants low in IU per level of uncertainty of the vignettes. For low uncertainty vignettes, again almost everyone chose to report all the results (96.3%, 26/27), whereas fewer participants chose to report all high uncertainty vignettes (55.6%, 15/27). These proportions were significantly different, ꭓ^2^ (1, *N* = 27) = 9.09, *p* = .001. There were also fewer participants to report all moderate uncertainty vignettes (74.1%, 20/27) than participants reporting all low uncertainty vignettes (96.3%, 26/27). This difference in proportions was significant, ꭓ^2^ (1, *N* = 27) = 4.17, *p* = .031. The proportion of participants reporting all moderate uncertainty vignettes (74.1%, 15/27) and the proportion of participants reporting all high uncertainty vignettes (55.6%, 15/27) did not differ significantly, ꭓ^2^ (1, *N* = 27) = 2.29, *p* = .125.

Table 3 shows the number of participants reporting everything versus not reporting everything for the participants high in IU per level of uncertainty of the vignettes. Three exact McNemar tests determined whether the differences in proportions were significant. For low uncertainty vignettes, everyone chose to report all the results (100%, 24/24), whereas fewer participants chose to report all high uncertainty vignettes (62.5%, 15/24). These proportions were significantly different, ꭓ^2^ (1, *N* = 24) = 7.11, *p* = .004. There were also fewer participants to report all moderate uncertainty vignettes (70.8%, 17/24) than participants reporting all low uncertainty vignettes (100%, 24/24). This difference in proportions was significant, ꭓ^2^ (1, *N* = 24) = 5.14, *p* = .016. The proportion of participants reporting all moderate uncertainty vignettes (70.8%, 17/24) and the proportion of participants reporting all high uncertainty vignettes (62.5%, 15/24) did not differ significantly, ꭓ^2^ (1, *N* = 24) = 0.17, *p* = .687. No difference was found between low IU and high IU in the pattern of results.
